# A Treatise on Fever

**Published:** 1861-07

**Authors:** 


ART. II.—*A Treatise on Fever; or Selections from a Course of Lectures on Fever.* Being part of a Course of Theory and Practice of Medicine, delivered by ROBERT D. LYONS, Physician to Jervis Street Hospital, Dublin, etc. etc. Philadelphia: Blanchard & Lea, 1861. 8vo., pp. 362.

DR. LYONS states in his preface that he was "induced to publish this work on Fever, with a view to bring within the reach of the student and junior practitioner, in a convenient form, the more recent results of inquiries into the pathology and therapeutics of this formidable class of diseases." With this preliminary statement we entered on an examination of the work, expecting some additions to our knowledge of fever. In this expectation we have been disappointed. We are unable to perceive that any new light has been shed on the pathological conditions embraced under this name; nor have we received any improved views respecting the management of the forms of fever which are treated of. We say this with a feeling of disappointment, for we were far from entering on a perusul of the book with a disposition to seek for its demerits, and it would have been much more agreeable to us to have arrived at a different conclusion. It is but fair to say that, before examining the treatise, our intention was to prepare an analytical review of it; but from the manner in which the subjects are handled this would be a difficult task, and we cannot consider that the work has claims to the space which an elaborate review would require. In justice both to the author and our readers, we are bound to give some of the grounds for the disappointment which we have experienced.

To the pathology of fever fourteen pages are devoted. The author enunciates the views of Virchow and his colleagues of the Berlin school, as embodying the doctrine most consistent with our present knowledge. These views are concisely set forth in the following extract:—

"Fever, then, in the opinion of Virchow, essentially consists in an increase of temperature, which is caused by an increased consumption of organic material in the system, and appears to have its origin in certain changes in the nervous system. These changes may be considered to affect primarily the regulator or moderator functions of the nerves, and to be of a paralytic nature. It is probable that the vagi nerves, and we would add the sympathetic, are primarily if not chiefly engaged in the production of the febrile phenomena."

We do not find fault with the author for adopting these views, untenable as they certainly appear to us; but we do think he should at least have stated the views which are held by the majority of pathologists and practitioners at the present time; in other words, the humoral pathology of fever should not have been entirely ignored in a work designed for the student and junior practitioner. As the author does not discuss the subject, we shall not do so here; but we cannot pass by it without alluding to the evidence that "the fever-poison first invades the animal system through the channel of the nerves," which is afforded by a consciousness of the first impression of this poison on the nervous system. The author states that credible testimony to the reality of such an impression is not unfrequently met with, and declares that he was himself sensible of the time when he contracted typhus. We had thought that this fanciful notion was long since obsolete. To say that clinical observation is utterly opposed to the statement, would be gratuitous. Assuming it to be correct, the author was bound to offer some explanation of the fact, that after these primary impressions on the nervous system are made by fever-poisons, days, weeks, and even months may elapse before the morbid phenomena due to their action in the system become developed.

Dr. Lyons classifies fevers into *primary*, *irritative*, and *eruptive*. The primary fevers are the continued and the periodic—yellow fever being included among the latter. The eruptive fevers embrace small-pox, measles, scarlatina, and miliary or sweating fever. Erysipelatous fever does not find a place in either of the classes. Under the head of irritative fevers are enumerated gastric fever, gastro-intestinal remittent, and hectic. The latter, as the author states, "owe their existence to lesion of a well-defined kind in particular parts of the system, and are to be regarded as the constitutional expression of localized disease."

What the characters are which belong to gastric fever, and distinguish this from gastro-intestinal remittent, the reader is left to conjecture, for neither of the so-called irritative fevers is treated of. It is clear that any local inflammation, accompanied by febrile movement, might be included in the class of irritative fevers with as much propriety as the affections named. The author freely admits this when he says:—

"A gastric or a hectic fever has, in fact, the same relation to the localized pathological process which causes it, as the pyrexial or febrile state attendant on ordinary local inflammation, whether of the viscera or external parts, bears to the physical disease which lights it up in the system."

Why, then, we may ask, have the three affections named been selected to compose a group styled irritative fevers, since, according to the author's own showing, they are not essentially fevers, but local affections?

The eruptive fevers are not considered, the author, in his preface, stating that he "thought it best to deal only with those forms of the disease which are of most practical importance." It is to be inferred that, in the author's opinion, the eruptive are inferior in importance to the continued fevers.

Periodical fevers—intermittent and remittent—are also excluded. The author contents himself with the consideration of yellow fever, which he places under the head of remittent fever. He gives as a reason for this, "that there is no ground for regarding it as a disease *sui generis.*" Of course, then, it is neither more nor less than a variety of remittent fever. That this view has been entertained, and is still held by some, is not to be denied; but that it should be the deliberate opinion of one who has carefully studied the natural history of yellow fever, and the circumstances connected with its production and diffusion, so far as these are known, seems to us remarkable. We beg leave, with due respect, to commend to the author's perusal the learned work of Dr. La Roche, to which we observe not even a reference, in looking over the chapters devoted to yellow fever. To have written on yellow fever without making reference to the labors of La Roche, is, in itself, a fair subject for critical animadversion.

The larger portion of Dr. Lyons's work is devoted to the continued fevers. Thirty pages are occupied with the consideration of synocha or inflammatory fever. We confess ourselves to be one of those who had supposed this variety of fever to have been effectually eliminated from the nosology. We had hoped that these names had been consigned to the tomb of the Capulets. But here they are in a work designed for the student and junior practitioner. If either the student or junior practitioner can form a clear apprehension of the diagnostic characters of the variety of fever which the author thus designates, he has an advantage over us which we freely concede, and which certainly does not provoke any jealousy on our part. As varieties of synocha the author mentions synochus —another name which we had hoped was dead and buried. We have to thank the author for devoting to this variety but half a page. Relapsing fever he considers as another variety of synocha, and bestows upon its consideration six pages, without any allusion to the observations of Dr.

Jenner. He considers that in certain cases the primary seat of mischief is in the liver, notwithstanding he has distinguished this as one of the primary fevers, defining the latter to be fevers developed independently of any specific pathological lesion.

Under the head of typhous fevers, or typhosis, five forms of disease are included, viz.: 1. Typhus or camp-fever. 2. Typhoid or enteric. 3. "A state of the system in which, independently of the before-mentioned essential fevers, the pyrexial action which attends local diseases, such as bronchitis, pneumonia, and occasionally hepatitis, assumes all the characteristics of the low typhoid condition, with tendency to early sinking, collapse, and fatal issue." 4. The typhoid state supervening upon epidemic cholera. 5. The low typhoid condition following severe injuries, wounds, or surgical operations, and "upon such diseased processes as erysipelas, phlebitis, and purulent absorption." It would be an insinuation against the intelligence of our readers, which we do not care to indulge, to point out the nosological inconsistency of classing the third of these varieties with typhus and typhoid fever. The author, indeed, says they are not essential fevers. The same inconsistency, although somewhat less striking, applies to the fourth and fifth varieties. However, typhus and typhoid fevers are the only varieties treated of.

We could excuse, in a measure at least, the incongruities to which reference has been made in the foregoing remarks, if the reader were furnished with a clear and accurate account of typhus and typhoid fever. The work is deficient in this redeeming recommendation. The descriptive histories of these two forms of continued fever are vague, incomplete, and contain not a few palpable errors. That the author has seen cases of these two fevers in abundance is probable, but that he has observed them carefully by the bedside, and studied with attention the writings of Louis and other observers, the work does not afford intrinsic evidence.

It is not a matter of gratification to us to have formed such an opinion, but a due respect to candor and frankness requires us to say that, so far from regarding Dr. Lyons's work an important acquisition to our medical literature, it is calculated to confuse and embarrass the minds of those for whose benefit it was professedly prepared.

